# Effects of Inoculation with Stress-Tolerant Rhizobia on the Response of Alfalfa (*Medicago sativa* L.) to Combined Salinity and Cadmium Stress

**DOI:** 10.3390/plants12233972

**Published:** 2023-11-25

**Authors:** M. Cecilia Pacheco-Insausti, Ivana Tamara Ponce, Miguel A. Quiñones, Hilda E. Pedranzani, José J. Pueyo

**Affiliations:** 1 Department of Soil, Plant and Environmental Quality, Institute of Agricultural Sciences, ICA-CSIC, 28006 Madrid, Spain; ceciliapachecoin@gmail.com (M.C.P.-I.); ma.quinones@csic.es (M.A.Q.); 2 Laboratorio de Fisiología Vegetal, Facultad de Química, Bioquímica y Farmacia, Universidad Nacional de San Luis, San Luis D5700HOI, Argentina; ivanatamaraponce@gmail.com (I.T.P.); hildaelizz@gmail.com (H.E.P.)

**Keywords:** alfalfa, *Medicago sativa*, *Sinorhizobium meliloti*, salt, cadmium, stress, phytochelatin, homophytochelatin, proline

## Abstract

Agricultural soil salinization, which is often combined with heavy-metal contamination, is an ever-growing problem in the current era of global change. Legumes have a high potential for nitrogen fixation and are ideal crops for the reclamation of degraded soils. Alfalfa (*Medicago sativa*) is a valuable forage crop cultivated worldwide. Alfalfa plants fertilized with nitrogen or inoculated with a salt- and cadmium-tolerant *Sinorhizobium meliloti* strain were subjected to combined NaCl and CdCl_2_ stresses. Our results showed that inoculated plants presented higher aerial biomass than nitrogen-fertilized plants when they were exposed to salinity and cadmium together. To assess the mechanisms involved in the plant response to the combined stresses, superoxide dismutase and catalase antioxidant enzymatic activities were determined. Both increased upon stress; however, the increase in catalase activity was significantly less marked for inoculated plants, suggesting that other tolerance mechanisms might be active. Cd accumulation was lower in inoculated plants than in fertilized plants, which appears to imply that inoculation somehow prevented cadmium uptake by the plant roots. Expression analyses of several involved genes suggested that inoculation stimulated the biosynthesis of proline, phytochelatins, and homophytochelatins, together indicating that inoculated plants might be better suited to withstand combined salinity and cadmium stress effects.

## 1. Introduction

The rapid increase in the human population and its activities during the last century has led to the intensification of environmental impacts. Drought, extreme temperatures, salinity, and soil contamination by heavy metals constitute threats to environmental sustainability and crop productivity worldwide [1]. Soil contamination by heavy metals can have a deleterious effect on soil microorganisms and plants, representing a potential risk to animals and humans through different exposure routes [2,3,4]. Plants growing in heavy-metal-polluted soils represent the main entry into the food chain [5]. Salinization is one of the most crucial factors threatening agricultural land throughout the world. Irrigation practices, particularly with saline water, aggravate this problem, mostly in arid and semi-arid regions, where evaporation rates are often higher than rainfall [6]. Soil salinization reduces the rate of infiltration and decreases water availability for crops, affecting the productivity of large arable areas [7]. Sodium, chlorine, and boron ions accumulate to concentrations high enough to cause toxicity. Salinization and heavy-metal contamination can act independently and often synergistically, which complicates diagnoses and solutions [8].

Developing crops with greater tolerance to stress conditions requires the broadening of the study field by focusing on combinations of stress factors [9]. The response of plants to a single type of abiotic factor can be quite different from their behavior in their natural environment, where several stresses occur simultaneously. Many recent studies have shown that halophytes are better adapted to cope with abiotic stresses—including heavy metals—than salt-sensitive plants [10,11,12,13]. The high metal tolerance of halophytes is strongly linked to plant stress responses that include their antioxidant systems [14], the production of osmoprotectants such as proline, and the scavenging of free radicals [15].

Studies in different plant species report that cadmium (Cd) is highly phytotoxic and causes germination and photosynthetic inhibition, imbalances in nutrition, alterations in antioxidant metabolism and hormone levels, growth reduction, and, eventually, plant death [16,17,18,19,20]. Cd contributes to the generation of reactive oxygen species (ROS) [21], which are considered the main source of damage to plant tissues [22]. The primary defense against ROS includes superoxide dismutase (SOD) enzymes that catalyze the dismutation of O_2_^−^ (superoxide) to O_2_ and H_2_O_2._ Then, catalases and peroxidases detoxify H_2_O_2_ [23].

Legumes are particularly well suited to colonize moderately contaminated or nutrient-deprived soils [24]. Most species develop nitrogen-fixing nodules by establishing symbiosis with soil rhizobia. The inclusion of nitrogen-fixing plants appears to be a good strategy for stabilizing vegetation and facilitating ecosystem development by increasing the nitrogen content of the soils and boosting vegetation cover [25]. Since nitrogen availability is a limiting factor for plant growth and development, the ability of legumes to establish symbiosis and fix N_2_ provides them with a distinct advantage over other plant families. Symbiosis allows them to achieve adequate nutrition according to their productive capacity, as they are not largely dependent on the availability of nitrogenous nutrients in the soil [26]. Symbiosis confers legumes increased tolerance to various abiotic stresses, including heavy-metal stress [27,28]. Nevertheless, saline conditions and heavy-metal contamination may limit nitrogen fixation by reducing rhizobial viability, nodulation, nodule number, size, and dry weight [29,30]. The use of bacterial inoculants might facilitate the establishment and development of legumes, even more so if stress-tolerant rhizobial strains are used [24,28].

The use of forage legumes with good biomass production and soil coverage that are able to live in saline soils contaminated by heavy metals appears to be a potential approach to exploiting affected marginal soils. *Medicago sativa* (alfalfa) is grown as a forage crop on more than 30 million ha worldwide in a wide range of soil and climate conditions [31]. Alfalfa is the forage species most commonly used as bovine feed because of its low production costs, high quality (digestibility and protein content), and regular presence during the year. The spread of alfalfa cultivation is supported by its high biomass yields in dry matter (DM) ha^−1^. Its excellent forage quality is capable of satisfying the nutritional needs of animals with high requirements [32], and its great adaptability to various environmental conditions facilitates its wide diffusion [33]. Dincă et al. [34] indicate that its extensive farming is driven by its valuable agronomic characteristics, such as perenniality, plasticity, and the capacity for the symbiotic fixation of atmospheric nitrogen when correctly associated with specific strains of *Sinorhizobium* (syn. *Ensifer*) *meliloti*. Thus, alfalfa fulfills a fundamental role in maintaining the structure and nitrogenous fertility of the soils in which it grows [35]. The cultivars available on the market offer a wide versatility in production, longevity, winter rest, and tolerance to abiotic and biotic stresses [36].

The aim of this work was to investigate whether inoculation with tolerant rhizobia had a positive effect on the tolerance of alfalfa to the combined effects of salinity and cadmium stress as compared to nitrogen fertilization. In our study, the individual salt and Cd stress conditions selected were not the highest that the plants could tolerate independently, as the combination of both stresses displayed additive negative effects on plant growth. To evaluate the effect of inoculation on the plant tolerance to the combination of salinity and Cd stress, the antioxidant defense system, the levels of cadmium accumulation, and the relative expression of genes involved in glutathione and phytochelatin biosynthesis and proline metabolism were analyzed in inoculated and N-fertilized plants under stress and control conditions.

## 2. Results

### 2.1. Identification of Rhizobia Tolerant to Salinity and Cd Stress

**Figure 1:**
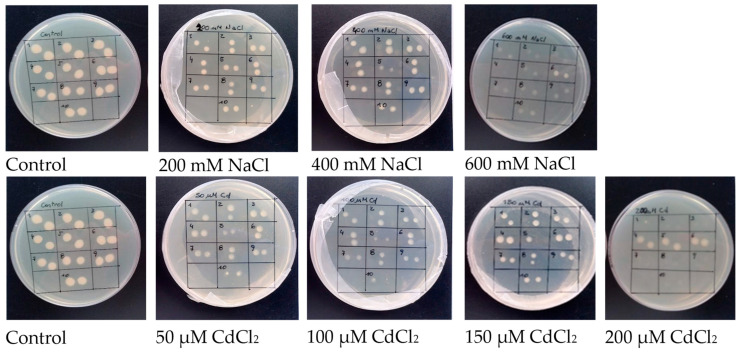
NaCl and CdCl_2_ sensitivity assay of 10 rhizobial isolates extracted from soil of the INTA EEA San Luis meteorological station. Each photo is labelled with the corresponding concentration of NaCl or CdCl_2_ used for that treatment. Assays were performed on agar plates in triplicate. Bacterial growth after 48 h is shown on one representative plate per treatment.

The ten rhizobial isolates studied presented a percentage of identity of 99% with the bacterial species *Sinorhizobium* (syn. *Ensifer*) *meliloti*. The NaCl and Cd tolerance of the rhizobial isolates was evaluated to determine their minimum inhibitory concentrations (MIC) (Table 1). Although all isolates showed growth at 600 mM NaCl, isolate 6 was the one that presented greater bacterial development (Figure 1). Isolates 1, 2, 3, 7, 8, 9, and 10 grew at 100 µM CdCl_2_, and isolates 4, 5, and 6 grew at 200 µM CdCl_2_ (Figure 1). Isolate 6 was selected as the most suitable strain with the highest tolerance to NaCl and Cd (Table 1).

### 2.2. Morphological Parameters

**Figure 2:**
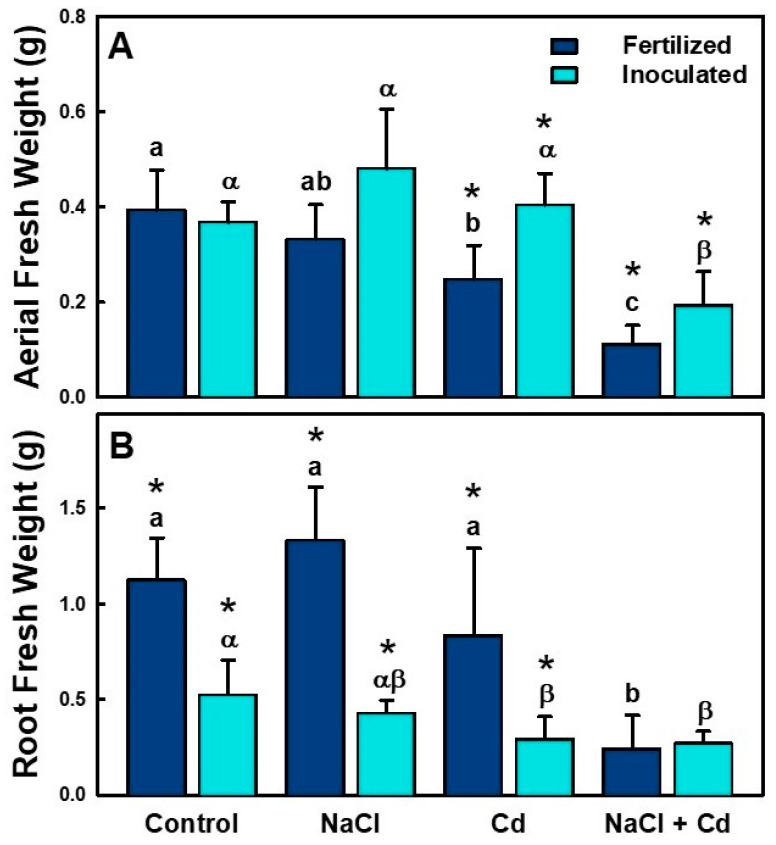
Aerial fresh weight (**A**) and root fresh weight (**B**) of *M. sativa* var. CW 660 plants inoculated with *S. meliloti* or fertilized with N under control conditions and NaCl (100 mM), CdCl_2_ (100 µM), and NaCl (100 mM) + CdCl_2_ (100 µM) stresses. Data presented are means ± SD (*n* = 5). Different letters denote significant differences between treatments in fertilized plants, different Greek characters denote significant differences between treatments in inoculated plants, and * denotes significant differences between fertilized and inoculated plants in the same treatment.

The morphological parameters AFW (aerial fresh weight) and RFW (root fresh weight) of alfalfa plants, either inoculated with *S. meliloti* (i) or nitrogen-fertilized (f), were evaluated to determine the effect of rhizobial inoculation on plant development under NaCl, CdCl_2_, and NaCl + CdCl_2_ stress conditions (Table 2, Figure 2).

Stress significantly affected the RFW of (f) and (i) plants (Table 2, Figure 2B). Both inoculation and treatment, as well as their interaction, had a significant effect on aerial and root fresh weights (Table 2). After 5 weeks, the AFW of (i) and (f) plants decreased significantly under the combined stress conditions but not under NaCl or Cd stress for inoculated plants. Inoculation favored the development of the plants, resulting in the higher biomass of (i) plants as compared to (f) plants (Figure 2A). The RFW of (f) plants was significantly higher than that of (i) plants for all treatments, except for the combined stress treatment, where no significant differences were observed between (f) and (i) plants.

### 2.3. Lipid Peroxidation and Antioxidant Enzymatic Activities

**Figure 3:**
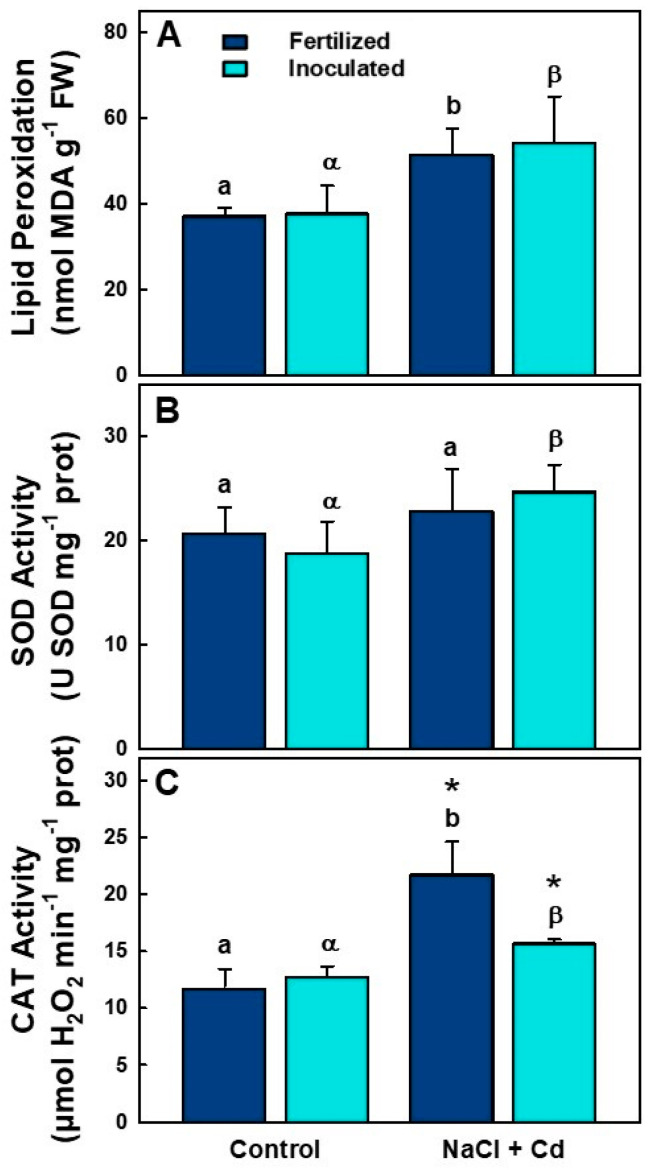
Lipid peroxidation (MDA) (**A**), superoxide dismutase (**B**), and catalase (**C**) enzymatic activities in *M. sativa* var. CW 660 plants under control and stress (NaCl 100 mM + Cd 100µM) conditions. Data presented are means ± SD (*n* = 5). Different letters denote significant differences between treatments in fertilized plants, different Greek characters denote significant differences between treatments in inoculated plants, and * denotes significant differences between fertilized and inoculated plants in the same treatment.

Lipid peroxidation (estimated as MDA accumulation) and the enzymatic activities of superoxide dismutase (SOD) and catalase (CAT) were evaluated to determine the effect of inoculation on alfalfa plants grown under stress conditions (Table 3 and Figure 3). Lipid peroxidation increased significantly under NaCl + CdCl_2_ stress in both (i) and (f) conditions (Figure 3A). Only treatment had a significant effect on lipid peroxidation (Table 3). The SOD activity of (i) plants increased significantly under stress, but that of (f) plants remained unaffected (Figure 3B). CAT activity increased significantly under stress conditions (Figure 3C). Furthermore, this enzymatic activity showed notably higher values in (f) than in (i) stressed plants (Figure 3C). Treatment had a significant effect on SOD activity, while inoculation, treatment, and their interaction had a significant effect on CAT enzymatic activity (Table 3).

### 2.4. Cadmium Accumulation

The concentration of Cd in leaves and roots was determined, and the translocation factor (TF), that is, the percentage of translocation of Cd from the roots to the aerial parts, was calculated. The accumulation of Cd was significantly higher in the leaves and roots of (f) plants than in those of (i) plants (Table 4). However, TFs were higher for (i) than (f) *M. sativa* plants (Table 4).

### 2.5. Real-Time qPCR Analysis

**Figure 4:**
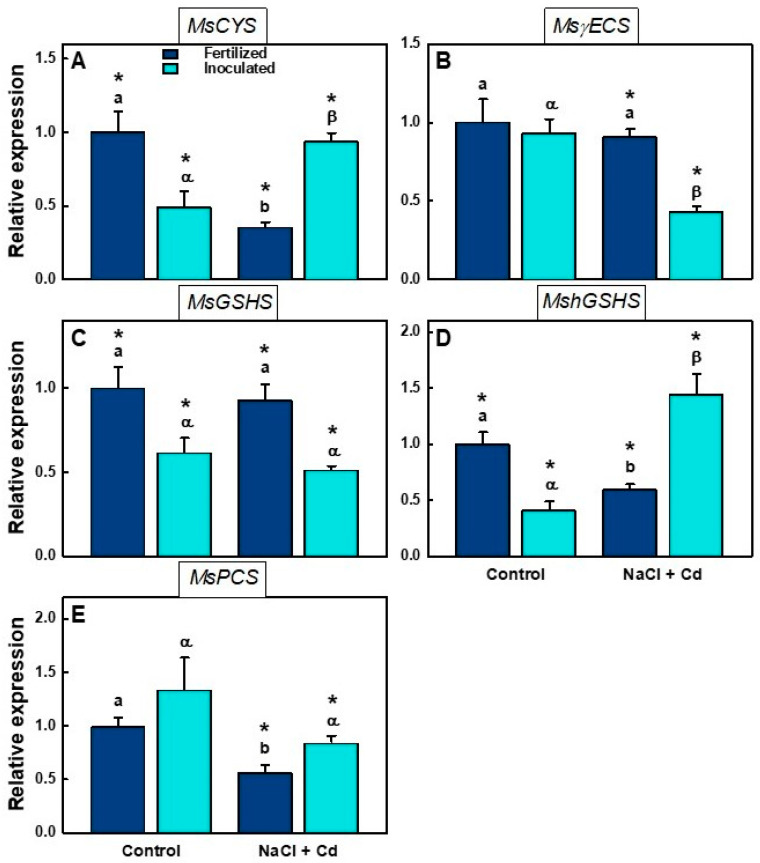
Relative expression of genes involved in glutathione and phytochelatin biosynthesis in inoculated (i) or fertilized (f) *M. sativa* var. CW 660 plants under control or stress (100 mM NaCl + 100 µM CdCl_2_) conditions. Data presented are means ± SD (*n* = 4). (**A**) *MsCYS*: cysteine synthase; (**B**) *MsγECS*: γ-glutamylcysteine synthase; (**C**) *MsGSHS*: glutathione synthetase; (**D**) *MshGSHS*: homoglutathione synthetase; (**E**) *MsPCS*: phytochelatin synthase. Different letters denote significant differences between treatments in fertilized plants, different Greek characters denote significant differences between treatments in inoculated plants, and * denotes significant differences between fertilized and inoculated plants in the same treatment.

**Figure 5:**
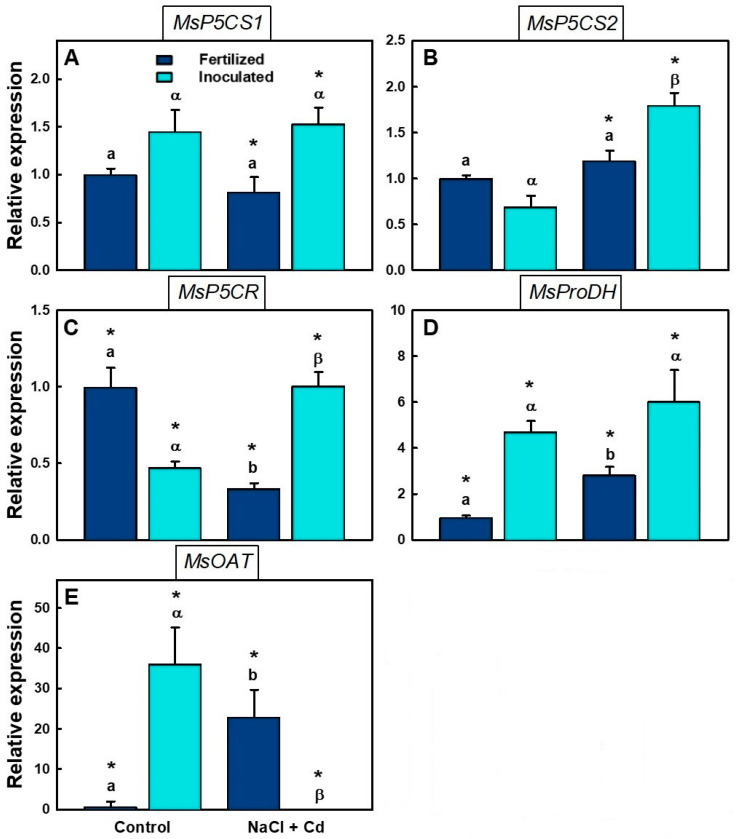
Relative expression of genes involved in proline biosynthesis in inoculated (i) or fertilized (f) *M. sativa* var. CW 660 plants under control or stress (100 mM NaCl + 100 µM CdCl_2_) conditions. Data presented are means ± SD (*n* = 4). (**A**) *MsP5CS1*: Δ1-pyrroline-5-carboxylate synthetase 1; (**B**) *MsP5CS2*: Δ1-pyrroline-5-carboxylate synthetase 2; (**C**) *MsP5CR*: pyrroline-5-carboxylate reductase; (**D**) *MsProDH*: proline dehydrogenase; (**E**) *MsOAT*: ornithine δ-aminotransferase. Different letters denote significant differences between treatments in fertilized plants, different Greek characters denote significant differences between treatments in inoculated plants, and * denotes significant differences between fertilized and inoculated plants in the same treatment.

The expression of several genes related to glutathione, homoglutathione, and phytochelatin (*MsCYS*, *MsγECS*, *MsGSHS*, *MshGSHS*, *MsPCS*) biosynthesis and proline metabolism (*MsP5CS1*, *MsP5CS2*, *MsP5CR*, *MsProDH*, *MsOAT*) were quantified in order to elucidate the effect of inoculation vs. fertilization on the response to combined salt and Cd stress in alfalfa (Table 5, Figure 4 and Figure 5).

Glutathione and phytochelatin biosynthesis genes showed, in general, a reduction in their expression under stress (Figure 4). However, there were some exceptions. *MsCYS* expression decreased significantly in (f) plants, while it increased significantly in (i) plants in the presence of NaCl + Cd (Figure 4A). The expression of the genes *Ms*γ*ECS* and *MsGSHS* decreased under stress conditions but was also significantly affected by inoculation (Table 5, Figure 4B,C). *MshGSHS* expression decreased in (f) plants under combined stress but increased in (i) plants (Figure 4D). A reduction in the expression of phytochelatin synthase (*MsPCS*) was observed under stress conditions. This decrease was significant only for (f) plants (Figure 4E).

Regarding proline biosynthesis, the expression of the different genes analyzed varied with treatment and inoculation (Figure 5). *MsP5CS1* expression did not change significantly upon stress (Figure 5A). *MsP5CS2* expression increased when plants were stressed, but the increase was significant for (i) plants only (Figure 5B). However, the relative expression of both *MsP5CS1* and *MsP5CS2* was significantly higher in (i) stressed plants than in (f) stressed plants (Figure 5A,B). *MsP5CR* expression was significantly enhanced by stress in (i) plants, but it decreased in (f) plants (Figure 5C). *MsProDH* expression increased in both (i) and (f) plants, but the change was only significant for the latter ones (Figure 5D). In addition, the expression of this enzyme was significantly higher in (i) than in (f) plants in both treatments (Figure 5D). *MsOAT* expression was significantly affected by all factors. *MsOAT* expression increased enormously in fertilized plants exposed to salt and Cd, while it was completely repressed in inoculated plants (Figure 5E).

## 3. Discussion

The presence of salt in the soil solution reduces the ability of the plant to absorb water, and this leads to a decreased growth rate. When the amount of salt is excessive, large amounts of solutes enter the plant during transpiration, causing damage to the cells in the transpiring leaves, which can cause further reductions in growth [37]. NaCl, the most common salt in saline soils, has been reported to increase Cd uptake and accumulation in plants when grown in Cd-contaminated soils [38,39,40,41]. It has been suggested that the formation of chlorocomplexes increases the mobility of Cd in soils, with the subsequent increased uptake of complexed Cd by plant roots [42]. This may explain why, in our experiments, plants exposed to combined NaCl + CdCl_2_ stress had drastically decreased aerial and root fresh weights compared to plants exposed to NaCl and Cd separately (Figure 2). However, the combined treatment did not affect the survival of the plants, and they all remained alive until the end of the experiment. The fresh weight of both inoculated and fertilized alfalfa plants decreased under combined stress conditions. These results agree with studies on other species, such as *Atriplex halimus* L., whose growth drastically decreased after exposure to Cd + NaNO_3_ [10]. Experiments carried out on two wheat genotypes exposed to NaCl and Cd stress also led to a significant decrease in shoot production [43]. On the other hand, in hyperaccumulating halophyte species such as *Solanum portulacastrum* [44] and *Carpobrotus rossi* [45], the presence of cadmium favored the development of biomass. Therefore, these findings suggest that the effects of salinity on the accumulation of Cd by different species are mainly related to their tolerance to salt and their tolerance to Cd independently [45]. Although the plants were affected by the treatment, inoculation favored the development of the plants, increasing the aerial biomass of the inoculated plants relative to the plants fertilized with N_2_ (Figure 2A). Rhizobial interaction with the plant may regulate calcium, nitrogen, and phosphate nutrients, with a favorable impact on their uptake, resulting in the growth promotion of the associated plant and its tolerance to abiotic stress [46,47]. Inoculation with a tolerant *S. meliloti* strain has been reported to reduce the independent negative effects of cadmium and salinity on alfalfa root nodules [48,49]. Nodulated legumes have been shown to be more tolerant to salt stress [6,50] and have been proposed as promising heavy-metal bioremediation tools [51,52].

Salinity and Cd accumulation produce an imbalance between the production and elimination of reactive oxygen species (ROS) in plants. One of the main consequences is the increase in intracellular levels of ROS, which can cause oxidative stress [53,54]. ROS include radical and non-radical oxygen derivatives, all of which are responsible for inducing oxidative damage to lipids, proteins, carbohydrates, and DNA [55]. In this work, oxidative damage, measured as lipid peroxidation, was evaluated in *M. sativa* plants either inoculated or nitrogen-fertilized under NaCl + CdCl_2_ stress conditions. MDA levels and SOD and CAT enzyme activities were determined in alfalfa leaves. MDA increased under stress conditions in both inoculated and fertilized plants (Figure 3A). These results agree with the increase in MDA in varieties of *Medicago truncatula* plants sensitive to Cd [20]. The enzymes superoxide dismutase (SOD) and catalase (CAT) are relevant because they are responsible for the detoxification of O^2−^ and H_2_O_2_, respectively [56]. SOD and CAT activities generally increase in most plants when they are stressed, independently of the cultivar or inoculation, including alfalfa [57]. We could see this general behavior in our study (Figure 3B,C). Plant-associated rhizobia can impart some degree of plant tolerance to abiotic stress [58,59]. However, while lipid peroxidation and SOD activity were similar for (f) and (i) plants under stress, CAT activity was lower in inoculated than fertilized plants, suggesting less of a negative effect of the combined stresses on the inoculated plants (Figure 2A) and/or the presence of alternative mechanisms to cope with salinity and Cd stress.

Accumulation and exclusion are two basic strategies of plants that respond to high concentrations of metals [60]. The ability to accumulate metal in the aerial parts with respect to the roots can be illustrated by calculating the translocation factor (TF), which provides an indication of the metal mobility from the roots to the aerial parts. In accumulator plants, the translocation factor is greater than 1, while in excluder species, the translocation factor is usually less than 1 [61]. According to our results, both calculated TFs were slightly higher than 1, indicating the mobility of cadmium from the roots to the leaves (Table 4). Therefore, fertilized or inoculated *M. sativa* could be considered mild accumulator plants under the conditions of our experiments. Cadmium accumulation was lower in inoculated plants than in fertilized plants, which might imply that inoculation somehow prevented Cd uptake by the plant roots. Cd accumulation in the aerial parts was lower in inoculated than in fertilized plants, which might represent an advantage, as it might help reduce the entry of the metal into the trophic chain.

**Figure 6:**
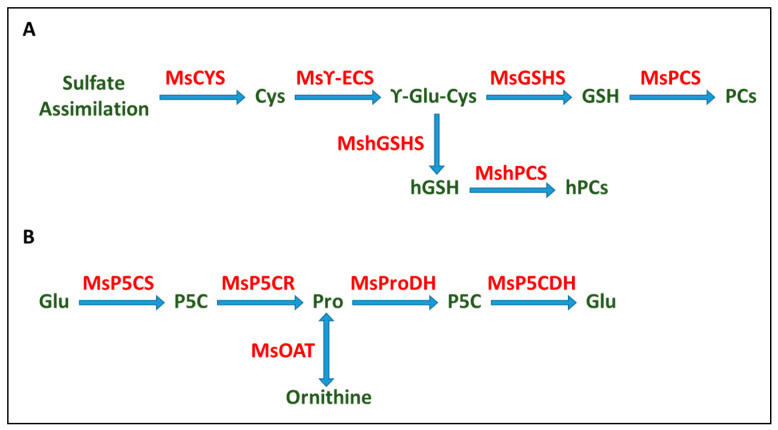
Schematic representation of glutathione and phytochelatin biosynthesis (**A**) and proline biosynthesis and catabolism (**B**) pathways. MsCYS: cysteine synthase; MsγECS: γ-glutamylcysteine synthase; MsGSHS: glutathione synthetase; MshGSHS: homoglutathione synthetase; MsPCS: phytochelatin synthase; *MshPCS*: homophytochelatin synthase; GSH: glutathione; hGSH: homoglutahione; PCs: phytochelatins; hPCs: homophytochelatins; MsP5CS: Δ1-pyrroline-5-carboxylate synthetase; MsP5CR: pyrroline-5-carboxylate reductase; MsProDH: proline dehydrogenase; MsOAT: ornithine δ-aminotransferase; Glu: glutamic acid/glutamate; P5C: 1-pyrroline-5-carboxylic acid; Pro: proline.

When Cd enters the cytosol, it activates the synthesis of several metal-binding compounds, such as glutathione (GSH), homoglutathione (hGSH), phytochelatins (PCs) homophytochelatins (hPCs), and proline (Pro) [62]. A schematic representation of GSH, PC, and hPC biosynthesis and Pro biosynthesis and catabolism pathways is shown in Figure 6.

Phytochelatin (PC) synthesis has been demonstrated to be a major metal detoxification mechanism in plants [63]. Basal phytochelatin synthetase gene (*MsPCS*) expression has been detected even without heavy-metal exposure, but it is strongly activated by metal ions [64,65]. In our study, there was no increase in *MsPCS* expression in plants under the combined stresses (Figure 4E). These results agree with those obtained with tomato plants, in which the PC content in leaves increased with exposure to 1, 5, and 10 μM Cd but decreased at higher concentrations of the heavy metal [66]. In alfalfa plants exposed to stress from CdCl_2_ and K_2_SiO_3_, the expression of *MsPCS* did not show significant changes with respect to the control [67].

In plants, glutathione (GSH) plays a key role as an antioxidant, chelator, and signaling molecule [68]. GSH is a precursor of phytochelatins (PCs) and a key constituent of the metal-scavenging machinery due to the high affinity of its thiol (-SH) group to metals. Inoculated plants increased their expression of *MsCYS* when exposed to stress. This could lead to higher levels of cysteine, which could activate GSH and PC biosynthesis pathways, despite the decrease observed in *MsγECS* expression. Also, in inoculated plants, an increase in *MshGSHS* expression was observed in stressed plants. In legumes, GSH can be replaced by homoglutathione (hGHS), and PCs can be replaced by homophytochelatins (hPCs), which are equally efficient in metal binding [69]. Our results suggest that inoculation has a positive effect on *M. sativa* plants, leading to the increased biosynthesis of PCs and hPCs, which bind Cd^2+^ ions and immobilize them in vacuoles. *Rhizobium* inoculation has also been reported to enhance copper tolerance by regulating phytochelatin-biosynthesis-related gene expression in alfalfa [70].

Proline (Pro) can be synthesized directly from glutamate (Glu) in a reaction catalyzed by Δ1-pyrroline-5-carboxylate synthetase (P5CS) or from ornithine (Orn) by ornithine aminotransferase (OAT). However, it is not always clear which pathway is involved in increased proline biosynthesis under stress conditions [71]. P5CS catalyzes the first step in Pro synthesis from glutamate [72]. The *P5CS* gene has two copies: *P5CS1* and *P5CS2*. Our study showed an increase in the expression of both *MsP5CS1* and *MsP5CS2* under combined stress in inoculated plants, which presented higher levels of both genes than fertilized plants. An increase in *MsP5CR* expression, which is involved in the second step in proline biosynthesis, occurred only in inoculated plants. These two facts suggest that inoculation activates Pro biosynthesis, thus providing a stronger defense against stress-generated ROS. Rhizobial inoculation has been previously described to promote proline accumulation under cadmium [73] or salt stress [50].

This is in agreement with previous studies by our group that show that transgenic *M. truncatula* plants that accumulate proline are more tolerant to salt and Cd stress [23,74]. *MsProDH* expression was higher in inoculated than in fertilized plants. However, stress did not affect its expression levels in any case, suggesting that stress did not increase Pro catabolism. *MsOAT* expression was very high in inoculated control plants, suggesting that Pro biosynthesis from ornithine could be relevant. This is in agreement with previous results [74] reporting elevated *OAT* expression in inoculated *M. truncatula* plants. The expression of *MsOAT* was diminished in inoculated plants subjected to combined salt + Cd stress. This result agrees with previous studies carried out in proline-accumulating *M. truncatula* plants exposed to salt or Cd [23,74]. Conversely, fertilized plants showed an increase in *MsOAT* expression when subjected to the combined stress; the same effect was observed for wild-type *M. truncatula* plants that did not accumulate proline when subjected to salt stress [74]. Although the genes and enzymes involved in proline biosynthesis have been well studied, the preferential use of Glu or Orn as a substrate is still unclear [75]. Some authors have reported that the preferred pathway depends on the stage of development, with the Orn pathway having a particularly crucial role in the developing seedling. Others, however, have documented that the route preference is species-dependent. This difference may also be related to the nutritional nitrogen status [76,77]. Even though some well-known mechanisms of tolerance to salt and heavy metal stress were investigated in this work and shown to be differentially activated in inoculated vs. fertilized plants, additional mechanisms of defense against abiotic stresses may also be involved.

In conclusion, the inoculation of alfalfa with a salt- and Cd-resistant *Sinorhizobium* (syn. *Ensifer*) *meliloti* strain had a protective effect against the combined stress of NaCl + CdCl_2_. Regarding aerial biomass, inoculation led to a significant increase when compared to chemical fertilization. All plants presented signs of oxidative stress after treatment. The lower increase in catalase activity observed for inoculated plants suggests that additional tolerance mechanisms might be activated. Cd accumulation in the leaves was lower in inoculated vs. fertilized plants, suggesting that inoculation somehow hinders cadmium uptake by the plant roots. Gene expression analyses showed that inoculation induced a coordinated response that suggests increased biosynthesis of phytochelatins, homophytochelatins, and proline, which together could imply that inoculated plants are better fit to cope with combined salinity and Cd stress effects.

## 4. Materials and Methods

### 4.1. Plant Material, Experimental Design, Treatments, and Growth Conditions

Alfalfa (*M. sativa*) var. CW 660 (Cal/West Alfalfas, Pergamino, Argentina) seeds were sterilized in 10% bleach for 15 min, rinsed with sterile distilled water (4 × 20 min), and left to imbibe at 4 °C for 30 min. Seeds were germinated in 1% agar in Petri dishes (25/19 °C, 16/8 h) for 48 h in the dark. Before sowing, seeds were inoculated by immersion for 1 h in the bacterial inoculum (10^8^ cfu mL^−1^; see Section 4.2). Non-inoculated control seeds were immersed in sterile water. Seeds were then transferred to 250 mL pots filled with sterile vermiculite. Plantlets were re-inoculated with 1 mL of a 10^8^ cfu mL^−1^ suspension of the corresponding bacterial strain, whereas 1 mL of water was added to non-inoculated plants. Plants grew in a growth chamber at 24/19 °C (day/night), with a 16/8 h photoperiod. The stress conditions were applied one week after sowing. All pots were watered with a volume of 30 mL three times a week. Non-inoculated plants were watered with Hoagland nutrient solution (NS) + 100 mM NaCl, NS + 100 µM CdCl_2_, or NS + 100 mM NaCl + 100 µM CdCl_2_. Inoculated plants were watered with NS with limiting N (5.05 g L^−1^ KNO_3_) supplemented with the same concentrations of NaCl and/or CdCl_2_. Control plants were irrigated with NS or NS with limiting N, depending on whether they were inoculated or not. A randomized block design was used considering two factors: (1) inoculation with *S. meliloti* or N fertilization and (2) stress application (100 mM NaCl + 100 µM CdCl_2_). The experimental unit was 1 plant, and 15 plants were used per treatment. After 5 weeks, morphological parameters were recorded, and tissue samples were frozen in liquid nitrogen and stored at −80 °C.

### 4.2. Isolation and Characterization of Rhizobia

Rhizobia were isolated from soil extracted from the INTA EEA San Luis meteorological station (UTM 20S: S275589 W6273722), San Luis, Argentina. Soil analyses were carried out at the ICA-CSIC Soil, Plant and Water Analysis Laboratory, Madrid, Spain (Table S1). For the isolation of the rhizobial strains, alfalfa trap plants were grown in vitro in test tubes with modified Jensen culture medium with nitrogen deficiency (1 g L^−1^ CaHPO_4_, 0.2 g L^−1^ K_2_HPO_4_, 0.2 g L^−1^ MgSO_4_·7H_2_O, 0.2 g L^−1^ NaCl, 0.1 g L^−1^ FeCl_3_, 8 g L^−1^ agar) [78]. A soil solution (1:10 p/v) was prepared and used to inoculate alfalfa trap plants, which were subsequently placed in a growth chamber under a photoperiod of 16 h (24 °C) light/8 h (20 °C) darkness. After 4 weeks, pink nodules were excised from the roots, sterilized with 10% bleach for 15 min, and rinsed with sterilized distilled water (4 × 20 min). A total of 10 isolates that grew in TY medium [79] and formed colonies with distinct size and color in agar plates were selected. To identify the isolates, partial sequencing of the 16S rRNA gene was performed. A 16S rRNA gene fragment was PCR-amplified from total genomic DNA using the primers fD1 (5′-AGAGTTTGATCCTGGCTCAG-3′) and rP2 (5′-ACGGCTACCTTGTTACGA-3′) [80]. The PCR products were examined by 1% TAE agarose gel electrophoresis and purified using the GFX™ PCR Gel Band Purification Kit (Amersham Pharmacia Biotech Inc., Erie, PA, USA). Sequence data were aligned and analyzed using BLAST (National Center for Biotechnology Information (NCBI) Bethesda, MD, USA). Samples were preserved in 30% glycerol at −80 °C until use. To analyze the strains’ tolerance to salt and Cd stress, agar plates with solid TY medium (bacto-agar 15 g L^−1^) were prepared with increasing concentrations of NaCl (0–800 mM) and CdCl_2_ (0–200 µM). All treatments were performed in triplicate. Then, 2 μL (~2 × 10^5^ cells) droplets of bacterial culture in the exponential phase were seeded. Plates were allowed to stand undisturbed until droplets were completely absorbed and then incubated at 28 °C. Colony growth was evaluated after 48 h. The minimum inhibitory concentration (MIC), that is, the lowest concentration at which bacteria were unable to grow, was determined [81].

### 4.3. Lipid Peroxidation and Enzyme Activities

Malondialdehyde (MDA) content was estimated using the 2-thiobarbituric acid reaction [82]. First, 1 g of fresh leaf tissue was mixed with 1 mL of 10% trichloroacetic acid and 1 mL of 0.67% thiobarbituric acid and then heated in a boiling water bath for 15 min. MDA was measured at 535 nm using a spectrophotometer. Lipid peroxidation was expressed as nmol MDA g^−1^ fresh weight.

Superoxide dismutase (SOD) activity was determined as previously described [83]. The amount of enzyme causing 50% quenching of the nitro blue tetrazolium chloride (NBT) photoreduction rate is defined as one SOD activity unit, and results are expressed in U mg^−1^ protein. Catalase (CAT) activity was measured by quantifying the amount of H_2_O_2_ decomposed, which was determined spectrophotometrically at 240 nm, as previously described by Redondo et al. [84], and expressed as μmol H_2_O_2_ min^−1^ mg prot^−1^.

### 4.4. Cadmium Content

The cadmium content in plant tissues was determined after acid digestion with HNO_3_/H_2_SO_4_ (initial volume ratio of 4:1) until the complete oxidation of the organic matter. The metal content in the extracts was quantified using atomic absorption spectrophotometry (AAS) with a Shimadzu AA 6650F system (Kyoto, Japan). Translocation factors (TF) of Cd were calculated according to the following equation [85]:

TF (%) = ([Cd]_aerial part_/[Cd]_root_) × 100

### 4.5. Real-Time qPCR

Total RNA was isolated using the trizol method (Invitrogen, Waltham, MA, USA), and RNA concentration was determined with a Nanodrop ND1000 spectrophotometer (Thermo Fisher Scientific, Waltham, MA, USA). RNA reverse transcription was performed using the Super Script RII Reverse Transcriptase kit (Invitrogen) and the oligo-d(T)18 primer. Primers3 2.6.0 software was used to design specific primers (Table S2). RT-qPCR analyses were performed using a 7300 Real-Time PCR Sequence Detection System Thermocycler (PE Applied Biosystems, Foster City, CA, USA). cDNAs were diluted 10-fold. Each reaction mix contained 1 μL of cDNA, 5 μL of SYBR Green PCR master mix (PE Applied Biosystems), and 4 μL mix of 2.5 μM specific oligonucleotides (forward and reverse) in a total volume of 10 μL. The amplification conditions were as follows: an initial denaturation cycle at 95 °C for 10 min, followed by 40 cycles at 95 °C 15 s and 60 °C 1 min. Data were analyzed using the System 7300 software v1.4.1 (Applied Biosystems). Relative gene expression was calculated according to the comparative CT method [86], taking into account the amplification efficiency of each pair of oligonucleotides, according to the following expression: ratio = [(E target) ΔCt target]/[(E endogenous) ΔCt], where E is the specific amplification efficiency of the oligonucleotides E = 10[−1/slope]. Fold changes ≥2 were considered significant. For each treatment, 4 biological replicates (4 cDNAs from 4 independent RNA extractions) were analyzed.

### 4.6. Statistical Analyses

Statistical analyses were performed with the IBM SPSS v. 27 software package (SPSS Inc., Chicago, IL, USA). The morphological parameters, MDA, antioxidant enzymes, and qPCR results were analyzed by multifactorial ANOVA. Significant differences among treatments were identified using the Tukey B test (*p* ≤ 0.05).

## Figures and Tables

**Table 1:** MIC (minimum inhibitory concentration) of the rhizobial isolates. Isolate number 6 (in gray) was selected for subsequent experiments.

Isolate No.	MIC	
	NaCl (mM)	CdCl_2_ (µM)
1	>600	<200
2	>600	<200
3	>600	<200
4	>600	>200
5	>600	>200
6	>600	>200
7	>600	<200
8	>600	<200
9	>600	<200
10	>600	<200

**Table 2:** Effects of the fixed factors inoculation and treatment and their interaction on the different parameters, analyzed by factorial ANOVA (*p* ≤ 0.05). Asterisks (*) denote significant effects. *, significant at *p* ≤ 0.05; ***, significant at *p* ≤ 0.001.

	Inoculation	Treatment	Inoculation × Treatment
AFW	***	***	*
RFW	***	***	***

**Table 3:** Effect of the fixed factors inoculation and treatment and their interaction on lipid peroxidation and antioxidant activities, analyzed by factorial ANOVA (*p* ≤ 0.05). Asterisks (*) denote significant effects. Ns, not significant; *, significant at *p* ≤ 0.05; **, significant at *p* ≤ 0.01; ***, significant at *p* ≤ 0.001.

	Inoculation	Treatment	Inoculation × Treatment
MDA	Ns	***	Ns
SOD	Ns	*	Ns
CAT	*	***	**

**Table 4:** Cadmium content in leaves and roots of *M. sativa* var. CW 660 plants and calculated translocation factors (TFs). Data presented are means ± SD (*n* = 5). Asterisks indicate significant differences (*p* < 0.05) between inoculated and fertilized plants.

Fertilization/Inoculation	Treatment	[Cd] (mg kg^−1^)		
		Leaves	Roots	TF
Fertilized	NaCl + CdCl_2_	11.81± 0.25 *	901.71 ± 16.25 *	1.31
Inoculated	NaCl + CdCl_2_	9.52 ± 0.02	477.54 ± 3.58	1.99

**Table 5:** Effects of the fixed factors inoculation and treatment and their interaction on gene expression, analyzed by factorial ANOVA (*p* ≤ 0.05). Asterisks (*) denote significant effects. Ns, not significant; *, significant at *p* ≤ 0.05; **, significant at *p* ≤ 0.01; ***, significant at *p* ≤ 0.001.

	Inoculation	Treatment	Inoculation × Treatment
*MsCYS*	Ns	*	***
*MsγECS*	*	**	*
*MsGSHS*	***	***	***
*MshGSHS*	***	Ns	Ns
*MsPCS*	Ns	*	Ns
*MsP5CS1*	**	Ns	Ns
*MsP5CS2*	Ns	***	***
*MsP5CR*	Ns	Ns	***
*MsProDH*	***	*	Ns
*MsOAT*	Ns	Ns	***

## Data Availability

The data presented in this study are available on request from the corresponding author. Original data collected and processed to obtain the results presented in this study are not publicly available due to their lack of interest.

## Supplementary Materials

The following supporting information can be downloaded at https://www.mdpi.com/article/10.3390/plants12233972/s1: Table S1: Physico-chemical soil analysis; Table S2: Primer sequences used for gene expression analysis.
