# Supplementary material for: Effects of Inoculation with Stress-Tolerant Rhizobia on the Response of Alfalfa (Medicago sativa L.) to Combined Salinity and Cadmium Stress
Source: Plants (Basel). 2023 Nov 25;12(23):3972. doi: 10.3390/plants12233972 (PMC10708420; doi:10.3390/plants12233972)
Supplement: Supplementary file 1 [file plants-12-03972-s001.zip › plants-2721525-supplementary.pdf]

Supplementary material

Table S1. Physico-chemical soil analysis

**Parameter**	
**pH**	8.34
**EC (μS cm^{-1})**	10140
**OM (%)**	1.57
**C (%)**	0.91
**N (%)**	0.124
**Clay (%)**	10
**Silt and sand (%)**	40-50
**Textural class**	Franco
**K (mg Kg^{-1})**	1294.4
**Ca (mg Kg^{-1})**	2547
**Na (mg Kg^{-1})**	4540
**Mg (mg Kg^{-1})**	602.6
**P (mg Kg^{-1})**	22.28
**Fe (mg Kg^{-1})**	24.7
**Mn (mg Kg^{-1})**	78.84
**Zn (mg Kg^{-1})**	1.3
**Cu (mg Kg^{-1})**	1.71
**Al (mg Kg^{-1})**	62.96

Table S2. Primer sequences used for gene expression analysis.

**Gene**	**Forward**	**Reverse**	**Acc. No. or locus name**
MsP5CS1	GAGGTCGATGTACGCGAGATG	CCTGAAGCCGTCTGGAACA	EU371644
MsP5CS2	GAGGGAATGGCCAAGTGGTA	TGGCAGCTCCTTGTGTGTATAAA	X98422
MsP5CR	ATCAAAACCGCCATCCACTCT	GACGCCGATGGACTCGAA	EX525188.1
MsProDH	CGACGCCGAGCTCAACTT	TGGGCACGTGCGAAAAG	AY556385.1
MsP5CDH	CTGCGGAGATCAGGTTCGTT	CCCAAGATGATTTCCAGGTACTG	AY556387.1
MsOAT	CTGGTGCTGAAGGTGTGGAA	TTCTTTTCATAACCCCACTTTCTTG	contig_4811
MsγECS	AAAGGATGGCTTGGAAAGAAGAG	CCACCTCAGCTACCGCATTT	AM407888.1
MsGSHS	GGTGTTCTTGAGAGGTTCCTTGA	TGCAAAGCATTCACGCATTT	AM411123.1
MsCYS	GCTGCCGCTGCAATTAAGA	CGACAACAATAAGCTTCCCAGAA	KY009966
MshGSHS	ACACGGCGTCAATTTTGATG	TCCCAAATTCTGAAATGACATGA	AM411122.1
MsPCS	CTGATCCTGCTTTCTGTGGTTTAG	CCAGGGTCAATAGCAAGAGCAT	AM407892.1
MtP5CR	GGGTTTCCGTGGAATACTGA	TCGAATTTTCGCTTAGAAATCA	Medtr7g090160

Accession Numbers (Acc. No.) and locus names were obtained from available genomic sequences at the National Center for Biotechnology Information (http://www.ncbi.nlm.nih.gov). *MsP5CS1*: Δ1-pyrroline-5-carboxylate synthetase 1; *MsP5CS2*: Δ1-pyrroline-5-carboxylate synthetase; *MsP5CR*: pyrroline-5-carboxylate reductase; *MsProDH*: proline dehydrogenase; *MsP5CDH*: Δ1-pyrroline-5-carboxylate dehydrogenase; *MsOAT*: ornithineδ-aminotransferase; *MsγECS*: γ-glutamilcisteine synthase; *MsGSHS*: glutathione synthetase; *MsCYS*: cysteine synthetase; *MshGSHS*: homoglutathione synthetase; *MsPCS*: phytochelatin synthase; *MtP5CR*: pyrroline-5-carboxylate reductase.
